# A UAV-Based Framework for Semi-Automated Thermographic Inspection of Belt Conveyors in the Mining Industry

**DOI:** 10.3390/s20082243

**Published:** 2020-04-15

**Authors:** Regivaldo Carvalho, Richardson Nascimento, Thiago D’Angelo, Saul Delabrida, Andrea G. C. Bianchi, Ricardo A. R. Oliveira, Héctor Azpúrua, Luis G. Uzeda Garcia

**Affiliations:** 1 Instituto Tecnológico Vale (ITV), Ouro Preto 35.400-000, MG, Brazil; regivaldo.carvalho@vale.com (R.C.); richardson.nascimento@vale.com (R.N.); hector.azpurua@itv.org (H.A.); lg.uzedagarcia@gmail.com (L.G.U.G.); 2 Control and Automation Engineering Department (DECAT), Federal University of Ouro Preto (UFOP), 35.400-000 Ouro Preto, MG, Brazil; 3 Computing Department (DECOM), Federal University of Ouro Preto (UFOP), Ouro Preto 35.400-000, MG, Brazil; saul.delabrida@ufop.edu.br (S.D.); andrea@ufop.edu.br (A.G.C.B.); rabelo@ufop.edu.br (R.A.R.O.)

**Keywords:** conveyor belt, idler rollers, thermography, UAV inspection, computer vision, maintenance

## Abstract

Frequent and accurate inspections of industrial components and equipment are essential because failures can cause unscheduled downtimes, massive material, and financial losses or even endanger workers. In the mining industry, belt idlers or rollers are examples of such critical components. Although there are many precise laboratory techniques to assess the condition of a roller, companies still have trouble implementing a reliable and scalable procedure to inspect their field assets. This article enumerates and discusses the existing roller inspection techniques and presents a novel approach based on an Unmanned Aerial Vehicle (UAV) integrated with a thermal imaging camera. Our preliminary results indicate that using a signal processing technique, we are able to identify roller failures automatically. We also proposed and implemented a back-end platform that enables field and cloud connectivity with enterprise systems. Finally, we have also cataloged the anomalies detected during the extensive field tests in order to build a structured dataset that will allow for future experimentation.

## 1. Introduction

The mining industry needs to transport large amounts of raw material. In addition to the iconic haul trucks, Belt Conveyor System (BCS) are one of mining’s most effective workhorses. In the bulk port where we conduct our study, more than 120 km of BCS are installed, demanding frequent inspection and maintenance due to their heavy use. A roller is the component used to support the belt which carries the material. Even though it is easy to assess the condition of one single roller, the task becomes grueling when more than two hundred thousand rollers spread over an area of almost 500 hectares need inspection. Furthermore, the effectiveness of the inspection relies completely on the inspector’s capabilities.

These facts combined increase the likelihood of inadequate inspections that may lead to future failures, production losses and damaged infrastructure. In this paper, we describe the implementation and field validation of the sensing and back-end modules of our solution. Using a teleoperated Unmanned Aerial Vehicle (UAV) equipped with a thermographic camera and offline signal processing techniques, the inspection procedure has been greatly improved, with quantifiable benefits in terms of precision, mobility, and productivity.

While the results of our extensive field-trials in the bulk port demonstrate that the concept works, we also discuss the main setbacks and conditions that affect the accuracy of the thermographic inspection in uncontrolled industrial environments; in summary, our main contributions are:

- The implementation and test of an UAV-based thermographic inspection procedure for belt conveyor rollers;
- A discussion about several factors influencing thermographic inspection of rollers;
- The creation of a labeled dataset of thermal images for supervised machine learning studies;
- A review of the main techniques to monitor conveyor belt rollers.

The rest of this paper is structured as follows: Section 2 introduces the challenges that mining companies face inspecting conveyor belt systems. Section 3 describes the failure mode of rollers, discuss the main techniques to monitor their condition and automated mechanisms of roller’s identification. Section 4 presents the layers and tiers of our proposed solution. Section 5 describes the field experiments, the challenges in real environments and discusses the results. Section 6 concludes the paper and outlines future work and research directions.

## 2. Problem Statement

BCS are extensively used for bulk material handling in different industries, particularly in mining. Ports are generally at the end of the mining process chain and rely on this equipment to transport a significant amount of material throughout different areas that include unloading points, stockyards and ship loaders.

Maintenance inspections are regularly performed on the different components of the BCS to ensure a safe and high-availability operation of this critical equipment. A small part of the components, like rolls, pulleys, take-up system, and driver unit are grouped in the head or the tail of the Belt Conveyor (BC), facilitating the installation of automated inspection systems. However, most of the components are dispersed over the extent of the conveyor system, making it harder to monitor or inspect them.

One of these components is the roller, responsible for supporting the belt and the transported material. The number of rollers is proportional to the extension of the BC. The conveyor configuration may vary, but a conventional BC has an idler with three carrying rollers spaced at roughly 1 meter. It means 3000 rollers per kilometer of conveyor requiring individual condition monitoring. At the bulk port where we conduct our study, the monitoring of more than 120 km of BCS is performed without instruments, with sensitive inspections, relying only on the experience of the maintenance team. The inspector walks on both sides of each BC and checks every roller for locked and damaged parts or abnormal noises. The current practice has several disadvantages, such as the poor quality of the inspection, the intensive use of skilled labor, and health and safety concerns related to hazardous environments.

**Figure 1:**
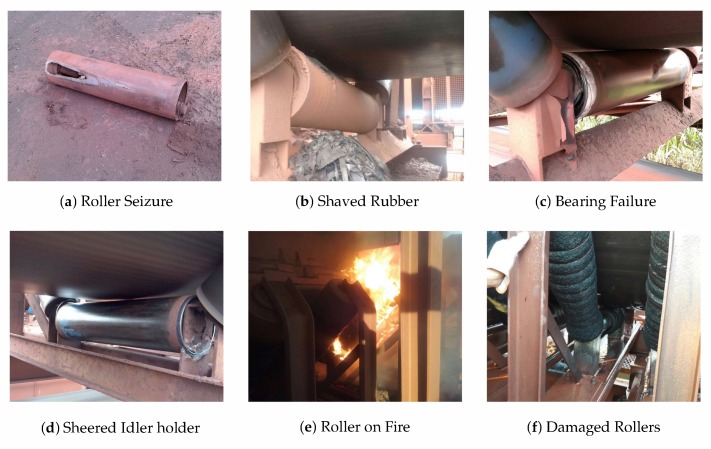
Rollers failures and its consequences: (**a**) Roller seizure that shaves the rubber of the BC, as demonstrated on (**b**). (**c**) A bearing failure affecting only the central roller. (**d**) Another bearing failure, this time, also affecting the idler holder. (**e**) An impact roller on fire and (**f**) rollers damaged by this event.

If the quality of the inspection is questionable, the consequences of an undetected defective roller are unpredictable. There may be no damage to the BC until the roller’s eventual replacement, but it may also overheat and set the belt on fire. In extreme circumstances, the fire may spread to other equipment in the port, what is a dangerous situation entailing high financial losses. Figure 1 demonstrates examples of defective rollers with different consequences.

## 3. Background and Related Work

### 3.1. Roller Structure and Failure Detection

The rollers are rotating machinery composed by two bearings mounted on a stationary shaft encased by a cylindrical surface (referred as cladding or shell) [1]. While a few defects affect the roller’s shell, the majority of them are related to the bearings.

As any rotating machinery, the rollers can be monitored by assessing the bearings’ condition based on three signals:

- **Acoustic:** Depending on the construction materials and internal characteristics, the bearing has specific acoustic emissions [2]. Despite the difficulty to isolate and process the frequencies of interest, an increase in amplitude or disturbances in the roller’s sound signature can indicate incipient bearing failure, emphasizing the predictive behavior of this signal [3].
- **Thermal:** When the roller is rotating, there is friction between its internal parts. A malfunction on the bearing increases the friction and so the temperature. Different thresholds can be used to evaluate the failure stage, but a 5 ${}^{\circ }$ C increase in comparison to adjacent bearings is already an indication of an early stage malfunction [4] or an uneven roller that wears faster.
- **Vibration:** Changes in the natural vibration frequencies evidence the malfunctions in the bearings. As with the acoustic signal, it is not trivial to isolate and process frequencies to diagnose failures based on vibration, but several techniques can be used [3]. It is also possible to classify the defect according to the vibration [5].

Next section discusses how the available techniques use at least one of the discussed signals to monitor the condition of conveyor belt rollers.

### 3.2. Condition Monitoring Techniques

**Figure 2:**
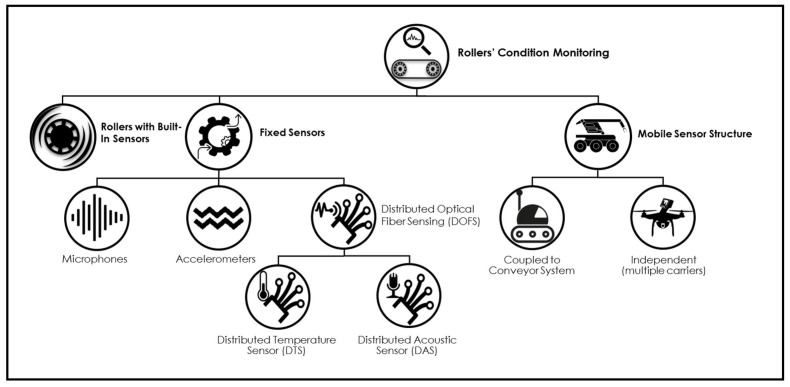
Sensor position and main techniques to monitor the condition of conveyor belt rollers.

Current techniques employ acoustic, thermal and vibration signals to assess the condition of the rollers. However, they differ on the type of the sensors used and how they are positioned (embedded in the roller, in the frame of the BCS or externally to the BCS). Figure 2 presents a diagram to classify the main solutions available.

The first group of solutions [6,7,8,9] encloses different types of sensors in the roller to capture thermal, vibration, and acoustic signals. The roller becomes an Industrial IoT device, with built-in ability to measure, classify and report abnormal conditions. However, all existing rollers must be replaced by smart models to achieve a fully continuously monitored BC, which it is not trivial due to the enormous amount of rollers in operations. Commercial solutions [10] are already available though.

The second group of solutions in Figure 2 is based on Fixed Sensors installed in the frame of the BCS. Li et al. [11] propose the installation of accelerometers to capture the vibration, Wavelet Packet Decomposition to isolate and identify the defective roller and Support Vector Machine to classify the failure. Similarly, Tan et al. [12] use neural networks and classifiers to recognize different failure types based on vibration signal. In turn, Jiang and Cao [2] capture acoustic signal with microphones and use Wavelet Transform to detect faulty behavior in rollers.

This kind of solution still uses a large number of sensors, proportional to the extension of the BC. An alternative is the use of Distributed Optical Fiber Sensing (DOFS), where an optical fiber cable act both as sensor and transmission media [13]. Two main optical fiber sensing principles can be used to monitor the rollers:

1. Distributed Temperature Sensing (DTS), which is based on Raman Optical Time-Domain Reflectometry (OTDR) principle [14]. Using this technique, Hu et al. [15] achieved a spatial resolution of 3 m with the uncertainty of 2 ${}^{\circ }$ C in a 10 km installation in an underground coal mine. The authors reported the meticulous calibration to insulate external factors in temperature measurement as the main drawback. Raman OTDR technology is still receiving improvements [16] and several commercial systems are based on it [17,18,19].
2. Distributed Acoustic Sensing (DAS) that is based on Rayleigh Coherent Optical-Time Domain Reflectometry (C-OTDR) principle [20] and relatively recent when compared to other DOFS technologies for roller’s condition monitoring [21]. There is ongoing research [22] and advanced-stage field tests [23], both with promising results. However, the launch of commercial solutions still depends on the ability to isolate and extract the condition from the frequencies issued by the bearings [24], since BCS and the environment where they operate are intrinsically noisy.

The third and last group of solutions presented in Figure 2 refers to Mobile Sensor Structures. One option is to have a sensor structure dedicated to a single BC, yielding a nearly continuous monitoring. An example is the work of [25], where the authors created a robot that moves inside the frame of the BC and capture infrared thermal images to assess the temperature of the rollers. The main drawback is that mandatory adaptations to the BC are generally not feasible.

Another possibility is having a detachable and independent sensor structure transported by different carriers, like ground robots [26], cable or rail drones, UAVs [26,27], and even human inspectors. This design adds flexibility and may also reduce costs, because a single sensor structure can be used to monitor multiple BCS. The obvious downside is the sequential (batch) monitoring. Other shortcomings are related to the carriers themselves. Human inspectors are slow, walking at 1.4 m/s [28]. Ground robots may face difficulties climbing ladders and accessing unpaved paths, while UAVs have indoor flying limitations.

Based on the discussed methods, we understand that the use of built-in sensors is not ideal for existing operations, due to the retrofitting requirements of more than 200,000 rollers in the port of the study case. Similarly, the use of fixed sensors, yet possible, is unfeasible to the extension of more than 120 km. DOFS is very promising, but the challenging calibration is the main weakness. As discussed above, mobile sensor structures also have their own handicaps, but they can be seen as a transition from manual inspection to online monitoring provided by built-in or fixed sensors.

Thus, we chose the UAV as a carrier, since it is: (i) not affected by terrain conditions; (ii) can fly at high speeds, which can reduce the inspection time and; (iii) are appropriate to be used in open areas, like the stockyards, where the greater extension of the BCs is located. To obtain the condition of the roller we chose the thermal signal. There are commercial thermal cameras for UAV and this signal is simpler to capture from a UAV when compared to acoustic and vibration signals, even though extracting relevant information is not trivial, as better discussed in Section 5.

To the best of our knowledge and disregarding patents [26,27], this is the first industrial-scale application of aerial thermography for rollers’ inspection. Other works linking thermography and UAV are focused on diverse applications, like crop management [29,30], power line [31,32] monitoring, and solar power plants inspection [33,34,35]. The works of [36,37] are examples of applications that use drones and machine learning algorithms to aid inspection processes. Next, we present the details and components of our solution.

## 4. Proposed Solution

**Figure 3:**
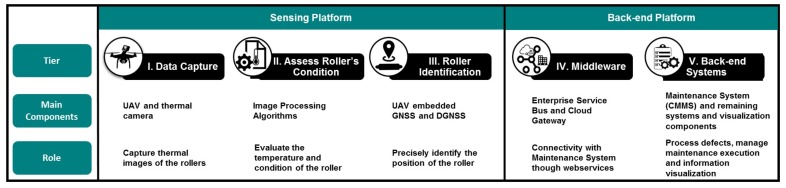
Main parts of the proposed solution to inspect conveyor belt rollers.

**Figure 4:**
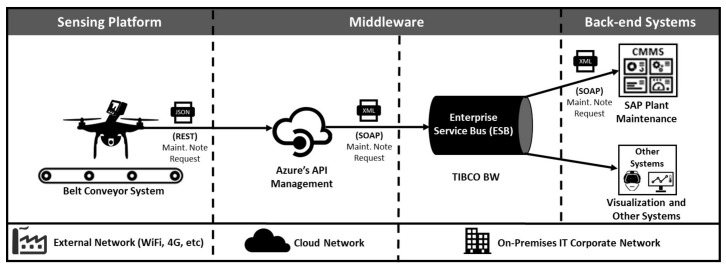
Middleware connectivity and protocol translation.

As demonstrated in the Figure 3, the solution is split into two layers: Sensing Platform and Back-end Platform. The former is composed of three tiers to capture the current state of the rollers and report defective conditions to Back-end Communication Platform. In turn, this layer enables the connectivity with systems in the organization and visualization tools (Figure 4), enabling the use of rollers’ condition information, particularly by the Maintenance System. The following subsections describe the tiers of each layer.

### 4.1. Sensing Platform

#### 4.1.1. Data Capture

This tier is responsible to provide the data used by remaining tiers of the solution. Our sensing platform follows the Mobile Sensor Structure approach, where we use a DJI Inspire I (UAV) to carry a DJI Zenmuse XT thermal camera. The UAV has a maximum autonomy of 18 min, a range of 5 km in interference-free environments and can maintain stable flights at the maximum speed of 21.9 m/s with wind speeds up to 10 m/s. In ideal situations, flying at 9 m/s, the UAV is able to inspect both sides of a 1.5 km BC in just 5.5 min, while an inspector walking at 1.4 m/s and spending merely 2 s to check each roller would take 5.6 h, almost a full day of work.

The DJI Zenmuse XT thermographic camera has a resolution of 640 × 512, a 19 mm lens and frame-rate of 9 Hz, enabling the capture of at least one frame each meter in a flight at 9 m/s. Moreover, it weighs 270 g, has a *Field of View* (FOV) of 32 ${}^{\circ }$ × 26 ${}^{\circ }$, *Instantaneous Field of View* (IFOV) of 0.895 mr, and features uncooled VOx microbolometers sensible to a spectral range from 7.5 to 13.5 μ m [38] that can be classified as Long-Wavelength Infrared (LWIR), ideal to measure temperatures from −20 ${}^{\circ }$ C to 650 ${}^{\circ }$ C.

Current local laws governing the use of UAVs do not allow fully autonomous flight. Therefore, a pilot must control the drone. The pilot is also responsible for taking photos and recording videos. These data are stored in the drone’s memory card and transferred to a computer after the flights. In future work, we will investigate the streaming of images and thermal information to the computer during the flight to enable online data processing.

#### 4.1.2. Assess Roller’s Condition

The first step is to find the bearings of the rollers in the images, which are the regions of interest. Next, we acquire radiometric data of each pixel inside this region and transform the image in a matrix of temperature values. Following, these regions are processed to extract the temperature of the roller and check its condition. This tier employs different image processing algorithms to this end. Each step of the method is better discussed in a real situation in the Section 5.1.3.

#### 4.1.3. Roller Identification

In this tier, Global Navigation Satellite System (GNSS) data stored in the image with a precision of 0.5 m in the vertical and 2.5 m in the horizontal are corrected using Differential Global Navigation Satellite System (DGNSS) data provided by base stations located in the port. Drone’s position is then compared with known georeferencing data of the BCs. With the distance of the UAV to the BC, altitude and longitudinal position, it is possible to obtain the position of a defective roller in the image. However, the integration and processing of each of this information is still under development and is not the scope of this paper to detail it.

### 4.2. Back-end Platform

#### 4.2.1. Middleware

The middleware tier is important to enable the connectivity between the Sensing Platform and Back-end Systems that consume information about the inspection. We focused on providing information to the Computerized Maintenance Management System (CMMS), since it is the most important system for maintenance planning, but this layer can be used as a bridge between the Sensing Platform and any system demanding rollers’ condition information. Such capability is possible due to the adoption of a Service Oriented Architecture (SOA) approach and an Enterprise Service Bus (ESB) to decouple the publisher (Sensing Platform) from the receiver (virtually any system).

As demonstrated in Figure 4, the middleware tier has two main roles: connectivity and protocol conversion. In the first role, it is used as a secure gateway between the corporative and external networks (4G, WiFi, etc.), where the Sensing Platform is connected to. We used Azure’s API Management [39] to expose a REST [40] API available through the Internet (cloud), so any request to this API is forwarded to the ESB in the corporative network through a VPN link.

The second role is protocol translation. The ESB already exposes webservices using Simple Object Access Protocol (SOAP) [41] to report defects in the CMMS. The middleware is used to convert Representational State Transfer (REST) requests (lightweight and ideal for mobile solutions) to SOAP.

#### 4.2.2. Back-End Systems

Information about defects is important to several systems within the organization, so the platform must report them as soon as they are identified on the field. Due to the criticality of the CMMS, we used it as an example of a back-end system demanding accurate information.

The CMMS globally used by the company is the SAP Plant Maintenance (PM). This system is responsible for managing the maintenance routine, including preventive and corrective work. The maintenance module is fully integrated with remaining modules of the Enterprise Resource Planning (ERP). This kind of integration streamlines company’s processes, but it also demands reliable information. The current practice, based on sensitive inspections, has two drawbacks regarding reliability: the quality of the inspection itself, since no instruments are used, and the manual input of inspection outcomes in the CMMS by inspectors. Types mismatch, the incompleteness of fault information, and inaccurate position of a defective roller are common.

Such occurrences can lead to rework in the maintenance routine. The defects reported by the inspectors are registered as maintenance notifications. Then, a maintenance planning team schedule and assign the work to an execution team, responsible to fix the problem, generally replacing the defective roller. If this group does not have the precise position of the defect, they will waste time re-checking rollers in the region reported or will simply cancel the work order, leading to human overhead and risks of not replacing a roller. Since such situations are real, a robust integration between the Sensing Platform and the CMMS is required.

## 5. Experiments and Results

**Figure 5:**
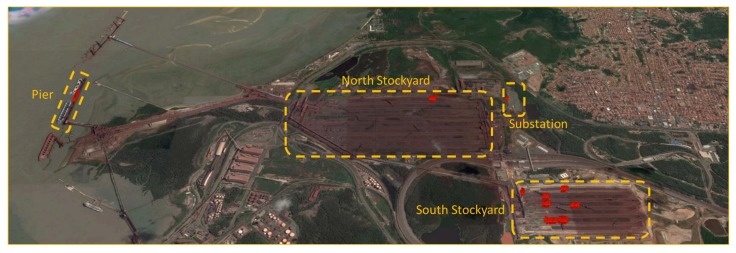
Overview of the test area with the Sensing Platform.

The field tests of our proposal were performed in a bulk port. We conducted tests at different times and on several BC to cover as many diverse situations as possible. The yellow dashed areas in Figure 5 represents the different locations where the tests were performed. Using the Sensing Platform, we captured 722 images represented as red dots in the image. After collecting this image database, we developed an algorithm in MATLAB to handle the computer vision tasks.

### 5.1. Basic Premises

To improve the accuracy in failure identification using thermography, some assumptions must be observed. Parameters like distance from the sensor to the rollers, sensor’s field of view, BC’s operation condition, camera’s characteristics, and production rate of the BCS at the moment of the inspection strongly influence in the measured temperature.

#### 5.1.1. Distance from the Camera to the Rollers

The distance from the camera to the object can hide elevated temperature values due to two factors. The first one is the influence of the atmospheric transmittance, but this factor can be adjusted in the camera’s parameters to minimize the error. The second one is that larger areas are represented by a smaller number of pixels as the distance to the object increases, therefore, the temperature values are locally equalized.

**Figure 6:**
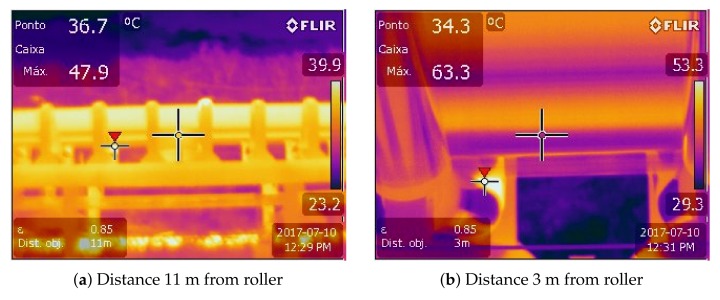
Distance influence in temperature measurement: (**a**) The photo was taken from 11 m, resulting in an image resolution of 1 pixels/cm ${}^{2}$. (**b**) Same roller with an image taken 3 m away and a 22 pixels/cm ${}^{2}$ image resolution. Both values for resolution are approximations.

The Figure 6 presents two images captured with the sensing platform at 11 m and 3 m. Both were captured in an interval inferior to 2 minutes, under the same operating conditions, emissivity of 0.85 and with the parameter of the camera distance to the object configured to match the actual distance. In the first image (Figure 6a) the maximum temperature value identified was 47.9 ${}^{\circ }$ C while the second image (Figure 6b) presents the maximum temperature of 63.3 ${}^{\circ }$ C. Despite the difference of 15.4 ${}^{\circ }$ C between the images, both detected the maximum temperature at the same point. The discrepancy can be explained by the image resolution, which is different at each distance. At 3 m, the camera resolution is approximately 22 pixels/cm ${}^{2}$, while at 11 m this value decreases to roughly 1 pixel/cm ${}^{2}$, meaning that the mean temperature of 22 pixels previously showed is now represented as a single pixel.

It is clear that a constant distance should be adopted during the thermographic inspection to avoid significant variations in the measured temperature. The UAV also needs to fly at a safe distance (5 m at minimum) from the BC to be able to safely react to unexpected wind direction changes. At this distance, a thermal camera with the resolution of 640 x 512 pixels and FOV of 32 ${}^{\circ }$ × 26 ${}^{\circ }$ (as the one employed in the tests) produce images with *Measurement Field of View* (MFOV) of 22.4 mm, which is sufficient to obtain reliable temperature measurements, since the roller has 203 mm of diameter.

Besides the distance, the UAV must also keep a fixed altitude to ensure a proper visualization of the central and lateral rollers. As the camera has a vertical FOV of 26 ${}^{\circ }$, at the distance of 5 m the vertical field of view will be of 2.3 m. It means that the UAV should keep an altitude of 2 m from the base of the BC. Under such conditions, the line of sight of the camera coincides with the superior part of the central roller, also positioned 2 m above the ground, an excellent condition of both central and lateral rollers.

During the experimental flights, the pilot kept the UAV at a distance of 5 m from the belt conveyor and at a height of 2 m from the belt conveyor base. By setting these distance parameters, we ensure that there are no significant variations in the measurement field of view. Therefore, temperature values measured throughout the flight do not need to be normalized.

These empirical tests aim to determine the optimum distance, height, and emissivity for the detection of defective rollers. Without this definition of distance parameters, there would be inconsistency in temperature values measured on different flights on the same belt conveyor. By adopting this strategy, in future work, we will be able to evaluate the evolution of roller operating temperatures over time and develop machine learning algorithms that can predict defects before they actually happen.

#### 5.1.2. Operation Condition and Production Rate

The thermographic inspection is based on the principle that the temperature rises according to the friction of defective parts of the roller, what only occurs while the BC is under operation. The rollers start to rotate when they are pulled by the conveyor belt, which is put in motion by driving rolls commanded by electrical motors generally positioned in the tail of BC [42,43]. If the BC is operating without any load, the temperature values can also be strongly affected. The nominal rates of the studied BCs are 8000 t/h or 16,000 t/h and the presence of this load at this rate significantly impact the rollers, particularly the defective ones.

**Figure 7:**
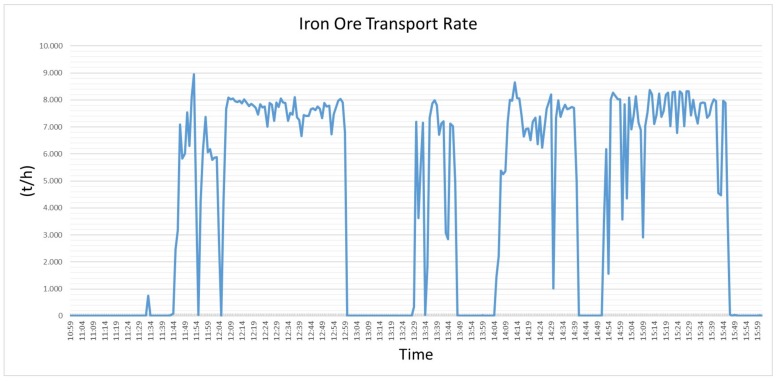
This chart shows how transport rate of one conveyor belt line can drop instantaneously and then recover the previous value of rate.

The Figure 7 presents the operation rate of one of the studied BC from 10:59 to 15:59 in the same day of Figure 6. During the assessed interval, the operation rate dropped significantly and immediately returned to the previous rate. In other time slots, as from 12:08 to 12:59, it operated at a rate with a standard deviation of 345.2 t/h, which is a small variation value to an iron ore operation. It also operated unloaded in different intervals, and 20 min was the maximum time it operated under this condition. Due to specific conditions on some BCs, like the slope of the belt conveyor and height, sometimes the inspector on the field does not know if the inspected equipment is operating with load or not. If the inspector collects the data while the BC is unloaded, as in one of the intervals demonstrated in Figure 7, the temperature values are unrealistic and real defects can be overlooked.

#### 5.1.3. Roller Failure Identification

A breakdown of a roller is not a big issue itself, but the consequences can be catastrophic. Fires, rips on the belt, and even work accidents are possible effects of a roller failure. Thus, while assessing rollers’ condition, false-negative detections should be minimized. In contrast, a false-positive detection does not have material and labor risks, but increases the maintenance costs with worthless work orders and should also be avoided.

On traditional inspection of BCS, even if the inspectors use advanced instruments as thermographic cameras, vibration or ultrasonic sensors, the quality of the inspection still depends on his/her personal experience and qualification. One of the objectives of our proposal is to improve the accuracy by highlighting the defects in thermal images, reducing and ultimately eliminating false-negative detection, regardless of inspector’s expertise.

**Figure 8:**
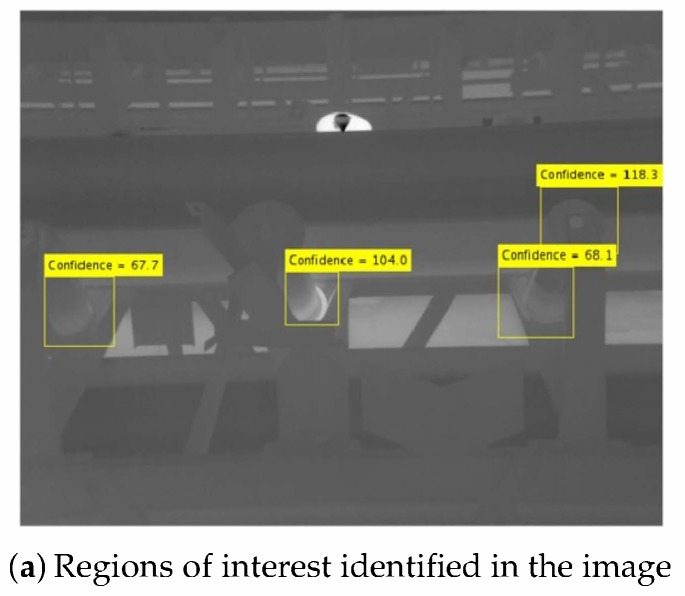

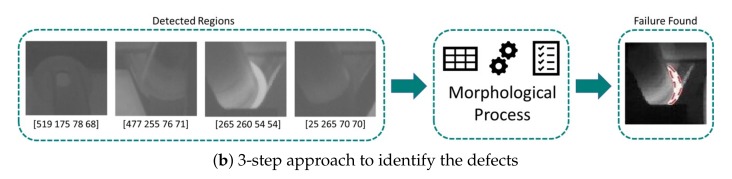
Assessment of defects on rollers.

To achieve that, we propose an approach with three steps. First, the UAV collects the thermal images following the assumptions discussed earlier. On the second step, the algorithm identifies the bearings of the rollers (regions of interest) in the images. Finally, by applying morphological processing of the radiometric data on the images, it is possible to extract the temperature and identify the defective rollers. The Figure 8 illustrates the steps in this approach.

### 5.2. Prior Object Recognition

Several approaches to identify objects in images are available [44], like the entire image search through a sliding window technique [45,46,47], segmentation methods [48,49], and other strategies using Convolutional Neural Network (CNN) [44] and Bag of Words (BoW) [50]. You Only Look Once (YOLO) [51] is a CNN-based method for object detection. It presents excellent results in terms of precision and is widely used in inspection scenarios [36,37], but requires a lot of computing power for real-time execution. The *Aggregated Channel Features* (ACF) method [52], which can be view as an evolution of the classical method of Boosted Cascade of Simple Features proposed by Viola-Jones [47], is another important machine learning technique for object detection. It presents a great balance of performance concerning the precision metrics and its real-time execution capability. According to experiments performed by Van Ranst et al. [53], the ACF method precision is only 8% lower than YOLO, while its execution can be seven times faster. Since processing speed and avoiding false-negative classification are crucial for rollers’ inspection, we chose the ACF method [52]. The training process is simple but very efficient in achieving false-negatives rates. The concept is to use a set of weak learners in each stage to form a strong classifier.

**Figure 9:**
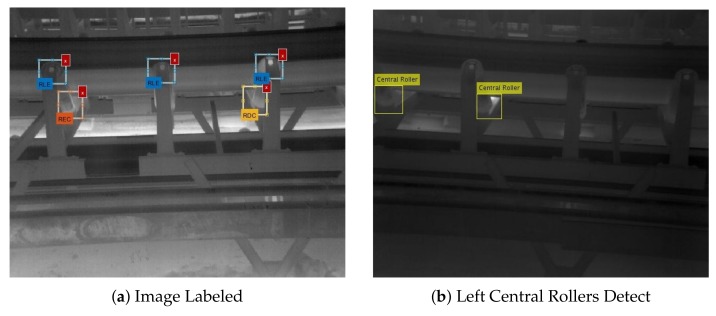

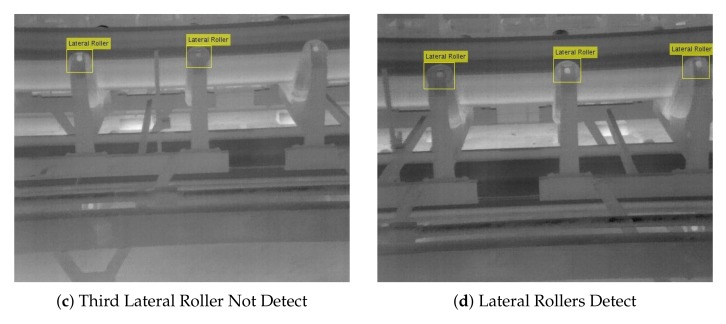
Training for Object Recognition: (**a**) Different regions of the rollers selected. (**b**) Left Central Rollers found using the trained detector. (**c**) Third Lateral Roller not found using the trained detector. (**d**) The Third Lateral Roller from Figure 9d, which is now the central lateral roller was found.

The ACF method proposed by Dollár and Appel uses several characteristic’s channels and a decision tree to form a robust and fast classifier. As illustrated in the Figure 9a, we used a dataset with 644 labeled regions to train the algorithm, split in three different areas: the tip of the lateral roller (labeled as Rolo Lateral (Side Roller) (RLE) with 324 regions), the intersection of the lateral with the central roller in the left (Rolo Esquerdo Central (Left Central Rollers) (REC), with 167 regions), and the intersection of the central roller with the lateral roller in the right (Rolo Direito Central (Right Central Roller) (RDC), with 153 regions).

The differentiation between REC and RDC were necessary to mitigate image recognition errors due to different visualization angles. For example, in the Figure 9b, we used only the detector trained to detect REC regions, so the RDC regions were not classified. In Figure 9c we applied the detector for the RLE and the third roller was not detected, what has been corrected on the subsequent image (Figure 9d), where all the rollers were detected correctly.

It is possible to control and tune several aspects of the training process, including the number of stages, which has a trade-off between the training time and the precision. Since the former were not a problem, we used 70 stages to optimize the latter. This step was repeated for each of the regions of interest. This roller detection algorithm was developed using Piotr’s Computer Vision MATLAB Toolbox [54].

#### Object Recognition Performance

To check the performance of the trained detectors, we used a dataset with 548 marked regions that were not used on the training. The dataset was split in 268 RLE, 167 RDC and 113 REC regions. We used the following metrics in the algorithm performance evaluation: *Average Precision* (AP), *Average Recall* (AR) and $F_{1}$ score. We also show the AP and *Maximum Recall* (R) metrics for each class of the database. We used the 50% *Intersection over Union* (IoU) threshold to identify a detection of a roller. The IoU metric is calculated between the areas of bounding boxes inferred by the model and bounding boxes of the ground truth.

**Figure 10:**
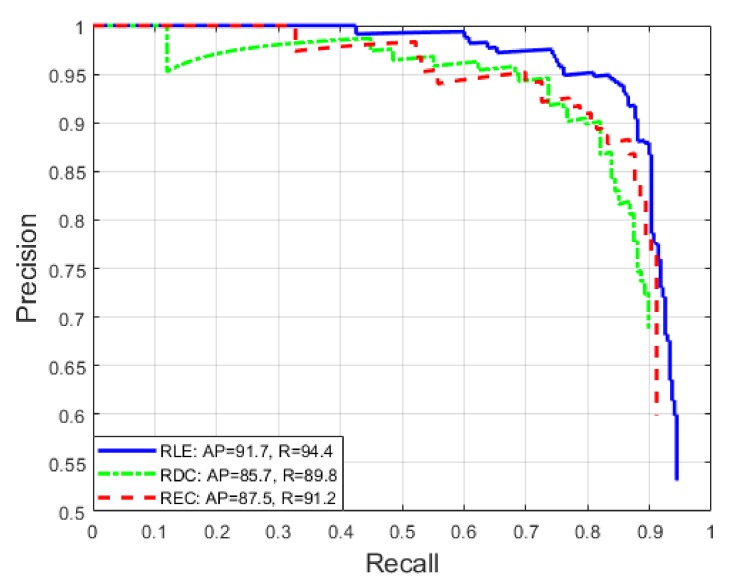
ACF algorithm evaluation.

Figure 10 shows the Precision x Recall curve, as well as the AP and R metrics, for each class of the database. The overall AP and AR metrics were 88.3% and 91.8% respectively, which gives our model an $F_{1}$ score of 90.0% .The *Overall False Negative Rate* (OFNR), which can be calculated from the AR, was only 8.2%, strongly impacted by the RDC regions, which had a OFNR of 10.2%, while the regions RLE and REC had 5.6% and 8.8% respectively. Since we have a spatial redundancy of 3 images, as demonstrated in Figure 9c,d, the probability of not detecting a region of the roller is only 0.06% ( ${\left(8.2\%\right)}^{3}=0.06\%$ ).

### 5.3. Failure Identification

Besides the identification of the areas where the temperature should be measured, the previous step also plays a role to eliminate undesired interferences. For instance, the reflectance is a serious issue of the thermographic inspections and, in some cases, can even indicate elevated temperatures in regions without defects. Since we know the properties of the rollers, we can properly set the parameters in the thermal camera and mitigate such external interferences.

**Figure 11:**
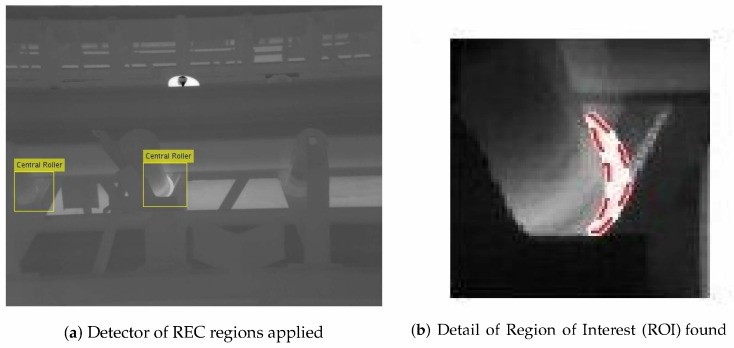
Defective roller found through the hybrid method: (**a**) ROIs selected. (**b**) Detail of the defective roller inside the selected region.

The Figure 11 illustrates how the method works. For this image we used only the detector for REC, therefore, central and lateral rollers were not identified, but two regions of interest were delimited (Figure 11a). Please note that there is a roller with a high temperature on the other side of the belt, but it is disregarded as we did not train it to detect opposite lateral rollers. In this context, it represents an example of an undesired interference, like the reflectance or a hot point, that was ignored as it is not part of the region of interest. Thus, only points inside the region selected are considered for temperature gathering by the morphological method, as demonstrated in the Figure 11b.

The morphological processing starts with reading the temperature data stored in each pixel within the bound boxes. In fact, the thermal images are false images produced from the temperature data collected. Therefore, we use the radiometric matrix of the regions of interest and use it to identify defective rollers. The morphological processing algorithm was developed using MATLAB’s Computer Vision Toolbox.

In our work, we define thermal defects as regions of a roller that have a temperature greater than 45 ${}^{\circ }$ C and an area greater than 19 pixels. These thresholds were empirically defined considering the UAV distance parameters relative to the belt conveyor used during the flights. As we kept these parameters fixed, the same threshold values were used for all images. The area threshold of 19 pixels represents an area of 384.7 mm ${}^{2}$ while the roller has a diameter of 203 mm and area of 32,349 mm ${}^{2}$, i.e., considering the distance parameters used, the defined area threshold represents about 1% of the total area of a roller.

We created a mask that marks as 1 the values greater or equal to 45 ${}^{\circ }$ C and as 0 the remaining values. It is also important to eliminate small regions that do not represent failures in the rollers or means error readings in one of the camera’s sensors. For this end, we used areas with size greater than 19 pixels, where each pixel is connected to 8 pixels that also have a temperature greater or equal to 45 ${}^{\circ }$ C. Finally, such regions can be plotted on the original image to highlight the defect to an inspector. The Figure 11b shows the result.

Although we have set these thresholds, our database does not contain pixel-level (segmentation masks) or roller-level annotations indicating whether a pixel or a roller contains or not a thermal defect (true positives or true negatives). This information will only be available after system deployment, which will enable data collection, annotation, and analysis by expert inspectors. Therefore, we use this definition of defects based on thresholds to assess the performance of our method.

#### Failure Identification Performance

Given the definition of a defect as a region with a temperature greater than 45 ${}^{\circ }$ C and an area greater than 19 pixels, the database does not present any failures. However, the morphological processing (MP) algorithm has identified six False Positive (FP) by using the thresholds of this definition. Thus, we realize that the morphological operation alone is not a good strategy for identifying failures.

Therefore, we need to observe the behavior of false positives and identify a method that reduces the number of occurrences to improve the results. FP occur near the edges of the bounding boxes and correspond to the regions of the belt rubber that have a high temperature. Thus, it is possible to eliminate most of these false detections by using a distance threshold. This threshold value is calculated individually for each bounding box and is a function of its width and height: t(w,h)=min(w/2,h/2), where *t* is the threshold, *w* and *h* represent the width and height of the box, respectively.

**Figure 12:**
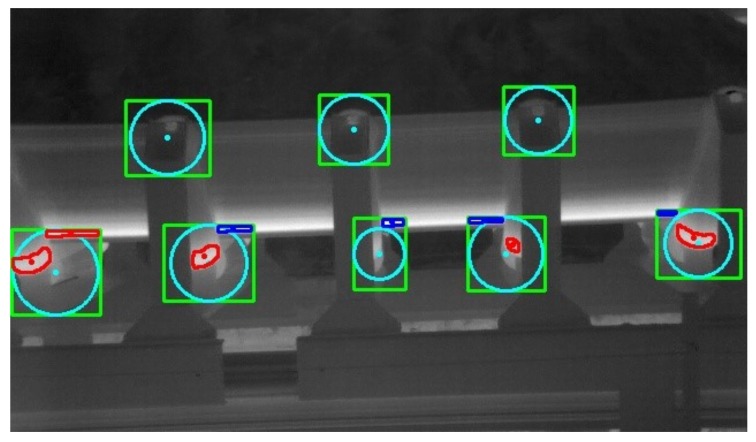
Defective regions found through the distance threshold approach.

We follow a two-step approach to apply this threshold. First, we calculate the distance between the centroid of the high-temperature region of interest (ROI) and the bounding box center. Then, we classify the ROI as a defect only if the distance is lower than the threshold. Figure 12 illustrates this procedure. The cyan point represents the bounding box center, and the cyan circle represents the area within the distance threshold. A high-temperature ROI is classified as a defect (red) if its centroid is within the circle. Otherwise, it is classified as a normal region (blue). Figure 12 also shows that this method reduces the number of false detections, but does not eliminate all of them. The roller on the far left of the figure has a region misclassified as a defect.

The failure identification using the distance threshold after the morphological processing (MP+DT) algorithm resulted in only one FP. A significant improvement compared to using morphological processing alone.

Despite this improvement, it is necessary to observe how this method impacts the identification of True Positive (TP). To increase the occurrence of defects in the database, we need to reduce the temperature threshold used in morphological processing. The temperature threshold value, which represents the defect in a roller, can vary according to several factors, such as the manufacturer, the type and quality of the production batch of the inspected rollers. Ideally, these temperature values should be updated continuously via experimentation by the maintenance team. For this reason, we decided to test the performance of the algorithm for two other temperature settings: 40 ${}^{\circ }$ C and 35 ${}^{\circ }$ C.

Considering the temperature threshold of 40 ${}^{\circ }$ C, the database contains 12 defects. The failure identification using only the MP algorithm generated a total of 12 TP and 45 FP, resulting in a precision of 21.05% and a recall of 100%. The identification of defects using the MP+DT algorithm produced 12 TP and only 2 FP, a result of 85.71% precision and 100% recall.

When analyzing these results, we noticed that the bounding boxes are centered on the rollers, and the true positives are close to the centers of their respective boxes. The distance threshold works so well because of this.

Considering the temperature threshold of 35 ${}^{\circ }$ C, the database contains 142 defects. The failure identification using only the MP algorithm generated a total of 142 TP and 155 FP, resulting in a precision of 47.81% and a recall of 100%. The identification of defects using the MP+DT algorithm produced 142 TP and 30 FP, a result of 82.56% precision and 100% recall. Table 1 summarizes the failure identification performance.

Table 1 shows that the algorithm based on the roller detection followed by morphological processing and the use of a distance threshold can identify failures with good accuracy and presents an $F_{1}$ score above 90%. Despite this, there are still some false positives that must be eliminated. An alternative to solve this problem is to use more advanced techniques, such as deep learning models for object and instance segmentation. However, it will demand more computing power than the method presented in this work.

## 6. Conclusions and Future Work

The semi-automatic inspection of rollers using a thermographic camera embedded in a UAV proved to be a very interesting approach to solve the accessibility and mobility issues associated with the inspection of these critical industrial components.

The expected productivity gain is significant, as the time taken to inspect a single 1 km long BC could drop drastically: while a skilled human inspector would require at least 2 h, a conservative estimate for the UAV-based system is 9 min. We also concluded that the same gain can be expected for the remaining 119 km of the BCS. The teleoperation of the UAV was feasible, even under challenging wind conditions at the pier. The identification of defective rollers was very effective as well, particularly during the night, providing sufficient information to visually identify different types of problems, such as locked rollers or bearing failures.

Despite the success, there is known room for improvement on multiple fronts. For example, in the Data Capture tier, flight stability could be improved with corrections from DGNSS base stations. Striking the right balance between dependability and expendability is another important issue. In other words, finding the right UAV and thermal camera combination in order to optimize capital and operational expenditures associated with this new inspection framework.

The automatic assessment of problematic rollers can also be improved. False-negatives numbers and false-positive detections can be reduced by using CNN-based deep learning models for object detection and segmentation instead of shallow learning techniques, as the ACF method used in this work. This is a trade-off between accuracy and execution time which will require more computing power.

Finally, the authors believe that truly autonomous flights can be a game-changer to deploy this framework on an industrial scale, even though local regulations could still prevent that. Active perception and robot vision are expected to play an important role in this application, such as the incorporation of acoustic sensors to increase the failures predictability. 

## Figures and Tables

**Table 1:** Summary for failure identification performance.

Temperature Threshold ( ${}^{\circ }$ C)	Number of Failures	Algorithm	TP	FP	Precision (%)	Recall (%)	$F_{1}$ Score (%)
35	142	MP	142	155	47.81	100	64.69
		MP+DT	142	30	82.56	100	90.45
40	12	MP	12	45	21.05	100	34.78
		MP+DT	12	2	85.71	100	92.31
45	0	MP	0	6	-	-	-
		MP+DT	0	1	-	-	-
